# Ecology and Evolution of Marine Fungi With Their Adaptation to Climate Change

**DOI:** 10.3389/fmicb.2021.719000

**Published:** 2021-08-27

**Authors:** Vinit Kumar, V. Venkateswara Sarma, Kasun M. Thambugala, Jun-Jie Huang, Xiang-Yang Li, Ge-Fei Hao

**Affiliations:** ^1^State Key Laboratory Breeding Base of Green Pesticide and Agricultural Bioengineering, Key Laboratory of Green Pesticide and Agricultural Bioengineering, Ministry of Education, Center for Research and Development of Fine Chemicals, Guizhou University, Guiyang, China; ^2^Department of Biotechnology, Pondicherry University, Puducherry, India; ^3^Genetics and Molecular Biology Unit, Faculty of Applied Sciences, University of Sri Jayewardenepura, Nugegoda, Sri Lanka

**Keywords:** *Aspergillus terreus*, ecology, eDNA, evolution, *Hortaea werneckii*, next-generation sequencing

## Abstract

Climate change agitates interactions between organisms and the environment and forces them to adapt, migrate, get replaced by others, or extinct. Marine environments are extremely sensitive to climate change that influences their ecological functions and microbial community including fungi. Fungi from marine habitats are engaged and adapted to perform diverse ecological functions in marine environments. Several studies focus on how complex interactions with the surrounding environment affect fungal evolution and their adaptation. However, a review addressing the adaptation of marine fungi to climate change is still lacking. Here we have discussed the adaptations of fungi in the marine environment with an example of *Hortaea werneckii* and *Aspergillus terreus* which may help to reduce the risk of climate change impacts on marine environments and organisms. We address the ecology and evolution of marine fungi and the effects of climate change on them to explain the adaptation mechanism. A review of marine fungal adaptations will show widespread effects on evolutionary biology and the mechanism responsible for it.

## Introduction

Global climate change affects the environment through shifts in mean temperatures and climate instability, along with other related changes such as ocean warming, stratification, acidification, eutrophication, and increased atmospheric carbon dioxide. Due to global warming, the average sea level is increasing by ∼3.2 mm per year (Stocker, 2014). Direct effects of climate change may alter the behavior, physiological functioning, and demography of organisms living in these environments (Hunter-Cevera et al., 2005). In marine environments, these alterations may affect species interactions and trophic pathways by propagating climate signals from primary producers to the degraders like marine fungi and affecting it in both bottom-up and top-down directions (Doney et al., 2012; Cavicchioli et al., 2019).

Marine fungi belong to different taxonomic groups and can be found colonizing and adapting to different substrates including driftwood, mangrove wood, roots, pneumatophores, seedlings, leaves of mangrove plants, soils and sediments in marine environments, seawater, and dead and decomposing animal substrata (Kohlmeyer and Kohlmeyer, 1979b; Hyde et al., 2000). Several species from marine habitats such as coastal oligotrophic and upwelling waters, deep-sea sediments, and sediments in anoxic zones have been reported (Damare and Raghukumar, 2008; Raghukumar, 2008; Gutiérrez et al., 2016; Lear et al., 2018). While filamentous higher marine fungi predominantly occur in coastal habitats such as mangroves and driftwood, the yeasts occur in open seas and deep-sea habitats. Moreover, the recent addition of molecular inputs has shown an increased discovery of novel species of marine fungi (Dayarathne, 2020; Jones et al., 2020; Devadatha et al., 2021). New species of *Aureobasidium*, *Cryptococcus*, *Candida*, *Exophiala*, *Malassezia*, *Rhodosporidium*, and *Rhodotorula*, have also been discovered from marine habitats such as hydrothermal vents and sub-sea floors (Overy et al., 2014; Grossart et al., 2016; Amend et al., 2019). Therefore, novel habitats, substrates, and the environment will remarkably assist in identifying new species that are adapted to them.

Adaptations of marine fungi to climate change can be understood through exploring the ecology and evolution of marine fungi from such extreme habitats. Up to now, several studies have reported ecology and evolution by utilizing morphological and molecular techniques (Sarma and Jeewon, 2019). As a result, novel fungal lineages have been found, based on a minimum of 3% nucleotide difference (Richards et al., 2012). Major adaptation events by fungi have also been explained, eventually providing new insight into the “white origin of fungi” (Rédou et al., 2015; Naranjo-Ortiz and Gabaldón, 2019). These works are widely regarded as pioneering which has led the experimental demonstrations on the ecology and evolution of marine fungi to expand at an ever-increasing rate. However, a comprehensive review of the adaptation of marine fungi to climate change, through their ecology and evolution is relatively rare.

We attempted to provide an overview of the adaptation of marine fungi to climate change through the above-mentioned topics. The importance of using molecular techniques to study the ecology and evolution of marine fungi is also discussed here. It may help scientists to improve current research practices to understand the purpose of adaptation that is important for evolution, where different ecological agents are expected to have different purposes. It may also allow non-professionals to better understand how marine fungi widen marine microbiological horizons. Information on fungal halotolerant genes or genes involved in adaptation to climatic changes will help in developing transgenic plants which may tolerate conditions like high salinity and high temperature.

## Ecology of Marine Fungi: Challenges and Concerns

Marine fungi play an important role in energy flow, exopolysaccharide complexes synthesis, and nutrient recycling. They intercede the cycling of dissolved organic matter and select appropriate decomposing techniques, such as comminution, non-enzymic chemical reactions, leaching, and volatilization (Sinsabaugh, 2005; Gessner et al., 2010). They perform denitrification in the hypoxic zones as reported from the Arabian sea (Raghukumar, 2008). Some marine fungi and fungi-like organisms degrade environmental pollutants in marine environments, e.g., *Thraustochytrids*, isolated from chronically polluted by oil spills in Goa, can degrade tar-balls (Raikar et al., 2001; Raghukumar, 2008).

Marine fungi produce various extracellular degradative enzymes, e.g., cellulases, ligninases, and xylanases (Raghukumar et al., 2004; Chi et al., 2007; Bonugli-Santos et al., 2015). Some enzymes are associated with nutrient-cycling in the deep-sea and they may be utilized as potential indicators of nutrient cycling processes, e.g., alkaline phosphatase in the deep sea plays a significant role in the recovery of inorganic phosphate by the catalysis of organic esters (Chróst, 1991). Fungi may be engaged in the production of humic aggregates in deep-sea sediments. The aggregate formation holds extracellular enzymes close to the secreting organisms and thus protectors and contributes to the overall sedimental nutrient cycling process (Damare and Raghukumar, 2008). However, it is difficult to comprehend the ecological functions of marine fungi (Gleason et al., 2012). Established roles include plant and algal waste degradation, chemical defense, pathogenicity, symbiosis, and contribution to various holobiont populations (Gleason et al., 2012; Balabanova et al., 2018). Following are some aspects of marine fungal ecology through different culture-dependent and culture-independent techniques.

### Culture-Dependent Techniques

Conventional methods of culturing marine fungi include (1) direct detection of fungal reproductive structures on natural samples by observing using stereomicroscope followed by single spore isolation, (2) culturing after surface sterilization of plant leaves or soft animal tissues and particle plating, (3) Baiting followed by culturing, and (4) dilution plating/direct plating (Raghukumar et al., 2010). Many marine fungi have been detected and cultured *via* direct detection and isolation (Kohlmeyer and Kohlmeyer, 1979a; Hyde et al., 2000; Jones et al., 2009, 2015). Some marine fungi are recorded with a higher percentage of occurrence with those encountered with ≥10% frequency indicating the “core group” of fungi at that site (Sarma and Hyde, 2001; Sridhar and Maria, 2006). Frequently occurring fungi are primarily studied by utilizing culture-dependent approaches. One such example can be seen from mangroves, where most of the fungi documented to date have been obtained based on culture-dependent methods (Sarma and Hyde, 2001). Using this type of method guarantees identification according to morphological, biochemical, or genetic characteristics (Jany and Barbier, 2008). Marine fungi produce sporulating structures such as ascomata, basidiomata, and conidia-bearing structures (anamorphic stage) in/on the substrates in which they grow actively in the form of hyphae. These substrates include mangrove wood, allochthonous wood, lignocellulosic materials such as coral, decaying leaves, macroalgae, cuttlebone of squids, and exoskeletons of crustaceans.

Culture-dependent approaches are a powerful tool with benefits in manipulating individual isolates, elucidating physiological properties, metabolic interactions among microorganisms and the surroundings, and accordingly provide statistics for their potential ecological roles in ecosystems (Otlewska et al., 2014).

Nonetheless, there are some drawbacks when using culture-dependent methods, e.g., culture-dependent approaches allow the isolation of only a small portion of the overall fungal diversity in an environment (Schabereiter-Gurtner et al., 2001; Jeewon and Hyde, 2007). Many metabolically active strains occur in the environment in the state of anabiosis, being viable but non-culturable (VBNC) and these are left out from being documented (Salma et al., 2013; Otlewska et al., 2014; Schottroff et al., 2018). Therefore, most of the fungal strains in environmental samples cannot be cultured and the culture-dependent methods provide only limited information on the biodiversity of microorganisms from that area (Poli et al., 2017). Moreover, culture-dependent methods are time-consuming due to long culture periods and elaborate culture techniques (Jany and Barbier, 2008; Stefani et al., 2015). To overcome these limitations, culture-independent approaches have recently been developed to explore and access the uncultured microbial community (Overy et al., 2019).

### Culture-Independent Techniques

Culture-independent techniques describe taxa more comprehensively than culture-dependent techniques. They are primarily focused on the use of next-generation sequencing (NGS), which along with phylogenetic data offers phenomenal evidence on relationships of species (Raimundo et al., 2018). It plays an important role in exploring marine fungal ecology, nutrient cycling, stress responses, and ecological niche construction (Dupont et al., 2007). NGS technologies have allowed unexplored marine ecosystems to be examined, e.g., hydrothermal vents, Mid-Atlantic Ridge, South Atlantic Ocean, where Ascomycota and Basidiomycota members were dominant, with various new phylotypes (Xu et al., 2017). High throughput sequencing (HTS) study of sediment samples from high Arctic fjord revealed 113 fungal OTUs by using ITS region, defined with a 97% sequence similarity cut-off (Zhang et al., 2015; Rämä et al., 2017).

Besides, genomic sequencing of model organisms from natural communities elucidates their biodiversity that promotes ecological structure, evolution events, taxonomic interactions, life history, and physiological biodiversity. For example, genomic sequencing-based characterization of a shared genomic element (nucleotide transporter) between *Rozella allomycis* and endoparasitic *Microsporidia* suggests that they share a common ancestor and *Rozella* leads a host-dependent lifestyle, where it depends on the host for essential metabolic genes (James et al., 2013).

Studies comparing culture-dependent and NGS techniques revealed wide variations in fungal community composition (Romão et al., 2017). Where, the culture-based approach reported low and variable levels of the species while the NGS methods (ITS1/ITS4 primers) revealed that the whole fungal population included *Purpureocillium lilacinum* in one study (Romão et al., 2017). Ecological roles of the dark matter fungi (DMF) in organic matter cycling have also been studied through environmental DNA sequences (SSU) (Grossart et al., 2016). DMFs are parasitic and saprophytic, they have not been cultured before and are missing from the taxonomy of the fungi.

Next-generation sequencing methods such as Illumina, Ion Torrent, and Pyrosequencing, are mostly used (Schlaeppi et al., 2016; Sanka Loganathachetti et al., 2017; Lendemer et al., 2019). Due to low cost, fast speed, and lack of cloning step in examining fungal diversity, Pyrosequencing was a preferred method (Lim et al., 2010). In a single run, it generates millions of short reads (300–500 nt) with a low error rate (Margulies et al., 2005; Buée et al., 2009). Despite this, it was discontinued in 2013, due to its non-competitiveness. In addition, the Illumina sequencing technology is widely used; though, it yields shorter reads than pyrosequencing, as this is improving rapidly (Snyder et al., 2010). In comparison to other NGS technologies, Illumina has a vast amount of sequencing depth. Further, Ion Torrent uses semiconductors, resulting in a higher number of sequence reads and faster processing times. It is being used to investigate fungal populations in mangrove and deep-sea soil compartments, where, *Aspergillus*, *Penicillium*, red-pigmented basidiomycetous yeasts, psychrotrophic fungi, and other uncultured deep-sea taxa were discovered (Nagano et al., 2017; Sanka Loganathachetti et al., 2017).

Large sequence reads are generated by technologies like PacBio, allowing complete lengths of barcode genes to be accessed (Kyaschenko et al., 2017; Tedersoo et al., 2017). It has been effectively used for the investigation of fungi metabarcoding. Despite this, researchers have been hesitant to use this approach due to its poor throughput, high error rate, and ever-changing bioinformatics methods (Tedersoo et al., 2017). However, advances in data processing algorithms applied to NGS data have reduced sequencing data preferences error rate, rendering them more accurate (Edgar, 2013).

Molecular methods such as DNA metabarcoding, in addition to NGS methods, allow to identify significantly greater taxonomic biodiversity within the samples (Stat et al., 2017). However, for effective fungal identification, the error rate is still too high (Li et al., 2019). Amplified rDNA restriction analysis, amplified ribosomal intergenic spacer analysis, denaturing gradient gel electrophoresis (DGGE), temporal temperature gradient gel electrophoresis (TTGE) and single-strand conformation polymorphism are some of the other techniques that can be used (Pang and Mitchell, 2005; Jany and Barbier, 2008). For example, DGGE was used to investigate fungal diversity in coastal areas where Ascomycota, Basidiomycota, Chytridiomycota, and novel environmental fungal clades predominated (Gao et al., 2010; Cury et al., 2011). However, since most research focuses on non-fungal microbial diversity, only a handful of these approaches have been used in the study of marine habitats (Raghukumar, 2008). As a result, the scope for using culture-independent approaches in marine habitats is enormous, and it holds great promise for revealing heretofore unknown fungal diversity (Raghukumar, 2017).

## Taxonomy and Evolution of Marine Fungi

**FIGURE 1:**
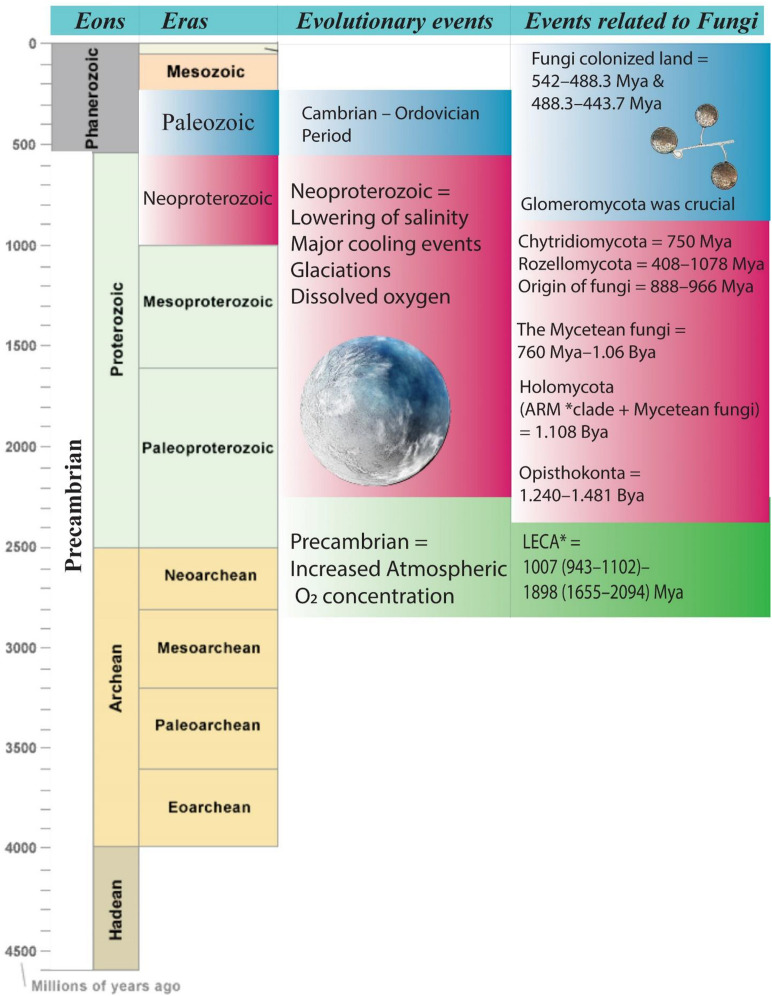
Schematic representation of evolutionary timeline related to fungi. *ARM, Aphelidomycota, *Rozella*, *Microsporidia*; *LECA, last eukaryotic common ancestor.

Fungi transitioned multiple times from marine to terrestrial environments, and vice versa (Amend et al., 2019). Numerous reports have recommended that fungi with plants were the first eukaryotes to inhabit the land, with mycorrhizal symbioses allowing this to happen (Lutzoni et al., 2018). Fungi inhabited land during the Cambrian to Ordovician periods, according to molecular dating (542–488.3 and 488.3–443.7 Mya) (Pisani et al., 2004; Dunn, 2011; Figure 1). The members in phylum Glomeromycota play a pivotal role in this colonization process (Hyde et al., 2017; Lutzoni et al., 2018). This has led to the theory that Glomeromycota members who lived in symbiotic relationships with cyanobacteria or algae eventually were symbionts of early land plants (Schüßler, 2002).

Studies proposed that marine ascomycetes diverged from many independent migrations of terrestrial and freshwater lineages to the sea (Jones et al., 2019). Where, bituniciates ascomycetes prefer tropical mangrove environments and unitunicates prefer temperate oceanic climates (Sharpe et al., 2015). Several freshwaters and terrestrial fungal genera, such as *Anthostomella*, *Didymella*, *Leptosphaeria*, *Lophiostoma*, *Massarina*, *Mycosphaerella*, *Passeriniella*, *Phaeosphaeria*, *Phomatospora*, *Saccardoella*, *Savoryella*, and *Trematosphaeria*, have marine members, indicating various land-sea transitions (Kohlmeyer and Kohlmeyer, 1979a; Hyde et al., 2000; Vijaykrishna et al., 2006; Jones et al., 2009; Suetrong et al., 2009; Sakayaroj et al., 2011).

Furthermore, Chytridiomycota and Rozellomycota (syn. Cryptomycota) are both marine early-diverging lineages. Chytridiomycota is flagellated fungi that diverged around 750 Mya (Chang et al., 2015). In terrestrial fungi that produce non-motile spores, the existence of flagellated zoospores was eventually lost (Sekimoto et al., 2011). Chytrids are parasitic organisms that infest phytoplankton and cyanobacteria. They may be saprobes or necrotrophs (Planktothrix) found mainly in nearshore and sediment samples (Comeau et al., 2016; Agha et al., 2018). Rozellomycota, on the other hand, has been found in anoxic marine environments, with an estimated divergence time between 408 and 1078 Mya (Grossart et al., 2016; Li et al., 2016; Tedersoo et al., 2018). Rozellomycota includes Rozella species as well as LKM-11 cluster sequences distributed in anoxic, aquatic, and marine habitats (Raghukumar, 2017).

What role do early diverging fungi play in the evolution of marine fungi, and what contribution do they make to the evolutionary system? The ARM (Aphelidomycota, *Rozella*, *Microsporidia*) clade includes early diverging communities such as Rozellomycota, Chytridiomycota, Mucoromycota, and Microsporidia, which are also pathogens of various other eukaryotes, such as amoebae, algae, and other fungi (James and Berbee, 2012; Tedersoo et al., 2018). The ARM clade can be dated back to the adaptation to intracellular parasitism (Corsaro et al., 2014). Aphelids, on the other hand, have more transitional characteristics than fungi and represent an earlier lineage in the holomycotan clade (Corsaro et al., 2014; Karpov et al., 2017). Since these classes have marine members, Aphelidiomycota (*Pseudaphelidium*), Chytridiomycota, and Rozellomycota would be important in understanding the evolution of marine fungi in the future (Comeau et al., 2016; Hassett and Gradinger, 2016; Karpov et al., 2017; Jones et al., 2019). However, they suffer from insufficient taxon sampling (Tedersoo et al., 2018).

ARM clade has a sister group called the Mycetaen fungi, which evolved between 760 Mya–1.06 Bya (Gingras et al., 2011; Beraldi-Campesi, 2013; Raghukumar, 2017; Jones et al., 2019). The Holomycota is the reference to both groups (1.108 Bya) (Jones, 2011; Raghukumar, 2017; Tedersoo et al., 2018). Mycetaen fungi and Metazoa (Animalia) are members of the Opisthokonta Division (1.240–1.481 Bya) and share a common ancestor that split during the Neoproterozoic era (Cohen et al., 2009; Parfrey et al., 2011; Doglioni et al., 2016; Tedersoo et al., 2018). The division of extant fungi and metazoans from a single ancestor is estimated in the early and mid-Neoproterozoic (Berney and Pawlowski, 2006; Sharpe et al., 2015). Whereas the age of the last eukaryotic common ancestor (LECA) is estimated to be between 1007 (943–1102) and 1898 (1655–2094) Mya (Eme et al., 2017; Figure 1).

During the Neoproterozoic era, salinity dropped with major cooling events, resulting in glaciations, and allowing dissolved oxygen (O_2_) into the ocean (Knauth, 2005). The Precambrian was responsible for increasing O_2_ accumulation in the atmosphere before the Neoproterozoic, but eukaryotes had only started to evolve during this period (Knauth, 2005; Doglioni et al., 2016). The key concern was the amount of dissolved oxygen in the water, which improved during Neoproterozoic. Based on divergence time analyses, the origin of fungi dates to 888 and 966 Mya, where Blastocladiomycota, Chytridiomycota, and Rozellomycota diverged around 750 Mya, indicating that early development of fungi occurred in the aquatic habitat (Sanderson et al., 2004; Chang et al., 2015; Tedersoo et al., 2018). However, this raises a debate about whether marine fungi originated from marine or freshwater habitats. To answer, we want to mention a few points, (1) salinity decreased in Neoproterozoic and O_2_ levels increased; (2) in the Precambrian era there was an increasing atmospheric level of O_2_, with atmospheric oxygenation, more diluted waters, such as lakes, rivers, and streams oxygenated well ahead of the ocean. Therefore, during the evolution in Precambrian and Neoproterozoic, lowering of temperature and salinity, and increased dissolved O_2_ were the main determinants that suggest the origin of the fungi in freshwater (James et al., 2006; Lücking et al., 2009; Raghukumar, 2017). The ocean allowed non-marine early fungi to transition to the current marine niche due to the significant decrease in salinity and a rise in O_2_. This claim thus indicates the origin of existing marine fungi in freshwater. However, the oceanic origin of existing marine fungi is still discussed (le Calvez et al., 2009; Raghukumar, 2017).

The concern about the fungal origin is most likely answered by high-throughput sequencing of genetic markers in several freshwaters and aquatic environments (Lear et al., 2018). As we learn more about the early-diverging fungal species, we would better understand the underlying mechanism of multicellularity, fungal colonization on land, and the origin of marine fungi.

## Consequences and Adaptation of Marine Fungi to Climate Change

In the marine ecosystem, almost all organisms depend on fungi, for the decomposition and recycling of carbon and minerals. To understand the consequences of climate change it is essential to understand the critical response of fungi toward it. In fungal growth, fruiting, and distribution in marine environments, climates play a dynamic and critical role. However, marine environments are getting progressively fragile due to various natural and anthropogenic stressors, including increased human population pressures, pollution, habitat loss, and degradation (Crain et al., 2008). Numerous biotic and abiotic factors influence the composition and distribution of marine fungal species (Hyde et al., 2000; Jones, 2011). Here we discussed several issues concerning the impact and adaptation of marine fungi to climate change.

## Increased Co_2_ Levels

Carbon dioxide is one of the most dissolved gas in seawater. In addition to salinity and aridity, greater concentrations of CO_2_ can be damaging to various marine fungi contributing to significant changes in vegetation (Sandilyan and Kathiresan, 2012). Increased degrees of atmospheric CO_2_ influence both the host and the associated fungal communities (Maček et al., 2019). Increased mycelium growth has been documented because of high CO_2_, including in arbuscular mycorrhizal fungi (Maček et al., 2019). Change has been observed in the community of Basidiomycetes on the coastal scrub oak forest soil after a 5-year treatment of soil with elevated CO_2_ (Klamer et al., 2002). This suggests, with the elevation in CO_2_ level, the composition of the soil fungal community changes significantly (Tu et al., 2015). However, the impact of CO_2_ on the fungal population from various environments should not be overlooked as it alters to some degree the community structure.

### Rising Temperatures

Temperature is a significant element affecting the worldwide distribution of marine fungi (Jones, 2000). Temperature fluctuations can significantly impact marine ecosystems and marine organisms, e.g., mangrove forests that are integral to marine ecosystems need a photosynthesis temperature of 28–32°C, leaf temperatures at 38–40°C can dramatically decrease the growth of mangrove trees, thus affecting net productivity. Increased temperature in fungal communities by 4–8°C leads to compositional changes that favor various classes of decomposers and promote the degradative succession of fungi (Venkatachalam et al., 2019). Species that are already under stress suffer the most due to habitat loss. Some obligate, as well as facultative marine fungi, follow the “Phoma-pattern” and prefer higher temperatures to grow (Ritchie, 1957; Venkatachalam et al., 2019), allowing mycetaen marine fungi to grow better at increased salinities and temperatures. This may lead to false-positive results while evaluating the fungal diversity of species involved in decomposition and restricted to temperature regimes (Chen et al., 2011; Xu et al., 2014).

### Rising Sea-Levels

Sea-level has a major influence on the climate and fungal diversity. Studies have shown the potential to disrupt marine ecosystems through rising sea levels. Strong cyclones may destroy mangroves by defoliation, uprooting, and tree mortality because of the accelerated increase in sea level. An increase in sea levels raises surface water and groundwater salinity by 1–33% in 25 consecutive years through saltwater intrusion which potentially influences the aquatic food web, food security, and expansion of salt-affected arable lands (Rahman et al., 2018; Ullah et al., 2021). Alongside this, the characteristics of soil sediments also change, which affects the fungal demography (Woodruff et al., 2013; Li et al., 2016; Tisthammer et al., 2016). Fungi which are dependent on host plants may also be affected when plants are affected in coastal ecosystems. Nevertheless, there have been no formal threats against marine fungi due to rises in sea levels. Studies relating the evaluation of marine fungal assemblages and environmental data with human development are thus required to forecast the response to climate change and identify directions for future coordinated management of marine ecosystems.

### Adaptation of Marine Fungi to Climate Change

Studies on the adaptive capacity of certain organisms to climate change and in extreme habitats could alleviate the detrimental impacts predicted by future climate change. The potential of some fungi for their phenotypic and genetic adaptation in response to extreme habitat and climate change has been acknowledged here. Several fungi have characterized themselves to adapt in environments with low water activity and high concentrations of toxic ions by complex molecular and cellular adaptation (Gostinèar et al., 2011; Gladfelter et al., 2019; Romeo et al., 2020). Spores of some marine fungi have developed strategies of sheath and appendages to attach, float, and adapt to a new environment. Similarly, some marine arenicolous fungi have developed subiculum to attach to sand grains and tolerate extreme conditions like high temperature, variation in salinity, and desiccation (Jones, 2000). These adaptation mechanisms and their effects are highly dependent on different levels of biological organization and follow a cascade of events (molecular and cellular, whole organisms, population, and community). Fungi isolated from hypersaline marine environments, deep-sea hydrothermal vents, and deep-sea sediments have different molecular and cellular mechanisms for adaptation, e.g., gene expression, high osmolarity glycerol (HOG) signaling pathway, melanization of the cell wall, composition and accumulation of ions, enzymes involved in fatty acid modifications, and plasma membrane composition (Turk and Plemenitaš, 2002; Kogej et al., 2006; Romeo et al., 2020). Fungi with these characters have been described for genera such as *Aspergillus*, *Cladosporium*, *Emericella*, *Eurotium*, *Hortaea*, *Trimmatostroma*, and *Wallemia*. Here we have discussed what molecular and cellular mechanisms determine the adaptation of *Hortaea werneckii* to marine habitat and how *H. werneckii* deals with climatic stressors such as high salinity (3–4.5 M) and temperature (Kejžar et al., 2015; Gunde-Cimerman et al., 2018; Romeo et al., 2020; Selbmann et al., 2020). Also, we have addressed the adaptations of *Aspergillus terreus* to the extreme conditions of a hydrothermal vent (Pang et al., 2020).

In yeasts (*Saccharomyces cerevisiae*), cells respond to the stress signals through sensing the environmental stimuli by mitogen-activated protein kinases (MAPKs) which ensure adaptation to the current environment (Plemenitaš et al., 2014). The sensor on the plasma membrane binds stimulus to the central MAPK cascade through membrane proteins, tyrosine kinase receptors, G-protein-coupled receptors, and histidine-aspartic phosphorylation sensors. The signal from sensors is passed to the MAPK kinase kinase (MAPKKKs), which phosphorylates the MAPK kinase (MAPKKs), activation of MAPKKs leads to the activation of MAPKs. MAPKs are then translocated to the nucleus to activate multiple factors for the adaptive transcriptional response. One such MAPK cascade called the HOG signaling pathway, has been characterized and conserved in *H. werneckii* (HwHOG) which acts as a survival and adaptation tool in hypersaline, marine, and deep-sea environments (Plemenitaš et al., 2014; Gunde-Cimerman et al., 2018; Romeo et al., 2020).

*Hortaea werneckii* is the most studied eukaryotic model organism in adaptive extremophiles, it can grow with or without salt and there is plenty of literature available on it. *H. werneckii* is placed in the family Teratosphaeriaceae (Capnodiales, Dothideomycetes), and can be found from beach soil, microbial mats, environments with low water activity, salty food, seawater, wood immersed in hypersaline waters, and rocks in tropical or subtropical coastal areas (Kejžar et al., 2015; Gunde-Cimerman et al., 2018; Selbmann et al., 2020).

However, *H. werneckii* is still not considered a marine fungus (Jones et al., 2015). Despite it has recently been isolated from different depths of the Mediterranean Sea and shallow hydrothermal vent as a common fungus (De Leo et al., 2019; Pang et al., 2019; Romeo et al., 2020). Pang et al. (2019) also considered that *H. werneckii* could be a marine fungus. Recently, based on observed phylogenomic differences between different strains, it has been found that the marine *H. werneckii* strains are derived by intraspecific hybridization, suggesting that marine strains are adapting and evolving in this environment (Romeo et al., 2020).

**FIGURE 2:**
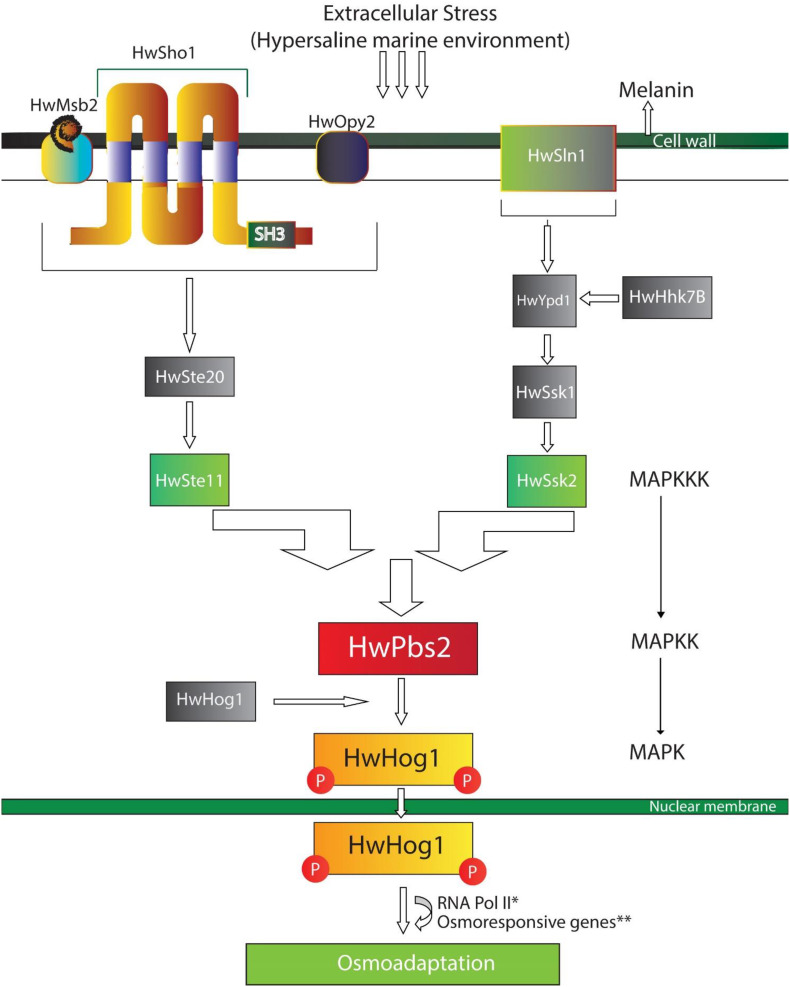
A schematic model of HOG – signaling pathway in *Hortaea werneckii*. Here, HwSho1 along with HwMsb2 and HwOpy2 interact with external stimuli (changes in salinity and osmolarity) and transmits the signal to HwHog1 (MAPK) mediated pathway through HwSte11 (MAPKKK) and HwPbs2 (MAPKK). In *H. werneckii*, the phosphorelay (HwSln1-HwYpd1-HwSsk1) interacts with cytosolic histidine-kinase (Hwhhk7) to transmit the external signal to HwSsk2. Signals from both sensors (HwSho1 and HwSln1) converge at HwPsb2, HwPsb2 activates HwHog1. Activated HwHog1 translocates to the nucleus and interacts with osmoresponsive genes and RNA Pol II for adaptation in fluctuating and hyperosmotic environments. *May or may not interact, **genes responsible for glycerol production, protein synthesis, amino acid metabolism, lipid metabolism.

Whole genome sequencing of a marine *H. werneckii* strains reported the up regulations and activation of genes related to stress-activated MAPK cascade (GO: 0051403), MAPKKK activity (GO: 0004709), cellular response to osmotic stress, heat, and oxidative stress (GO: 0071470, GO: 0034605, GO: 0034599), and regulation of mitotic cell cycle (GO: 0007346). Besides this, several Heat Shock Proteins (Hsps) were also activated such as HSP88, HSP78 mitochondrial, and HSP DnaJ (Romeo et al., 2020). Suggesting that cells of *H. werneckii* respond and adapt to fluctuating NaCl concentrations, heat, and extreme conditions in its environment by immediate responses, without requiring the synthesis of new proteins but modulate the pre-existing ones in metabolism and membrane transport mechanisms. Such modulation requires the activation of signal transduction pathways by stress signals (Kejžar et al., 2015; Gunde-Cimerman et al., 2018; Román et al., 2020). The combination of signaling pathways enables cells to resume growth and adapt to the conditions of marine and other extreme environments, leading toward their adaptation (Marchetta et al., 2018; Román et al., 2020; Romeo et al., 2020; Figure 2).

Similarly, in *A. terreus* (Aspergillaceae, Eurotiales); genes related to MAPKs and the HOG signaling pathway were up-regulated along with several other pathways, during intracellular osmotic balance and stress tolerance. Pang et al. (2020) investigated the growth of *A. terreus* at different pH, temperature, and salinity, mimicking the stressed environment, also the molecular adaptation of *A. terreus* was studied with the transcriptome analysis. At higher temperature, salinity, and low pH, *A. terreus* was able to grow optimally. This was validated by the transcriptome results, where, due to high temperature higher expressions of Hsps were observed along with that high temperature-induced reactive oxygen species (ROS), to counter this, genes related to catalases and superoxide dismutase were up-regulated (Abrashev et al., 2014; Pang et al., 2020).

Several proteins are associated with fungal reactions to acidic pH, such as up-regulation of phenylalanine ammonia-lyase (PAL), ATP-binding cassette transporters (ABC), and gamma-aminobutyric acid, while pH binding transcription factor (Pac C) and acetyl xylan esterase were down-regulated (Pang et al., 2020). To tolerate the salinity stress, genes related to arginine metabolism, HOG-pathway, MAPK, and linoleic acid were up-regulated, suggesting their involvement in intracellular osmotic balance. Pang et al. (2020), suggest that marine *Aspergillus* species are able to tolerate a range of environmental stress with the help of their stress-related genes and they could be a great source of such genes for transgenic studies.

Apart from these species, marine fungi such as *Aspergillus aculeatus*, *Microascus brevicaulis*, *Penicillium oxalicum*, and *Trichoderma harzianum* are also able to grow at different environmental conditions including high temperature and salinity, suggesting that they could also adapt to different changes in the environmental conditions (Jones et al., 2015; Pang et al., 2016, 2020).

## Conclusion and Future Prospects

1. Adaptation of marine fungi to climate change and extreme environment is an unending topic and there are more to be understood regarding it. In this review, we discussed how climate change leads to changes in the physicochemical properties of marine habitats, which alters the ecological structure, function, physiology, and population of individual species. Also, how certain marine fungal groups have adapted to these conditions. We have also discussed the molecular ecology and evolution of marine fungi and their origin based on the existing literature.
2. Cellular and molecular alterations due to climate change could be either as a response to the changing dynamics of the marine environment or due to the direct influence of stressors on the fungi. To counter stressors like osmotic and temperature fluctuations, *H. werneckii* utilizes HOG-pathway for osmoadaptation. Osmoadaptation includes adjustments in metabolism, cell surface properties, cell morphogenesis, growth and proliferation, and cellular protectant production, such as glycerol, erythritol, arabitol, mannitol. The regulation occurs either by activation and/or recruitment of specific transcriptional factors or associating with RNA polymerase-II, or both. *H. werneckii* produces melanin, which accumulates on the outer cell wall, forms a dense shield-like layer to protect the cell from UV. At optimal NaCl concentration, melanized cell walls help to retain glycerol in the cell.
3. Similarly, *A. terreus* isolated from shallow hydrothermal vents were able to grow at 45°C, pH 3, and 30% salinity. Along with this, genes related to stress tolerance were also shown to be up-regulated, suggesting the molecular adaptation of *A. terreus* to extreme conditions and environmental changes (Pang et al., 2020).
4. Marine fungi can adapt to the high salinity, temperature, and severe pH levels, which provides them with greater variety in biotechnological applications and offers an important biological advantage over terrestrial fungi. The understanding of the adaptation of marine fungi in extreme environments would help researchers to develop transgenic plants that can grow in such environments and provide greater flexibility during changing climatic conditions.
5. Studies on marine fungal ecology and evolution are rare, this could be due to (1) fewer marine fungal taxa were recorded than terrestrial habitats; (2) large geographical areas were still not explored; (3) some marine fungal taxa had recently been investigated with a lot of work yet to be carried out; (4) convergent evolution may have masked evolutionary relationships, and (5) a huge amount of marine water dilutes any evidence of environmental genetic material available. Through using molecular techniques with culture-based approaches, new environmental sequences from many marine environments will make a significant contribution to fungal diversity (Richards et al., 2012; Zhang et al., 2014).
6. There are continuing discussions about whether sequence-based species description can be accepted and recognized for taxon identification? (Hawksworth and Lücking, 2017; Hongsanan et al., 2018; Thines et al., 2018). In our opinion, the classification by sequence is not sufficient but may provide leads to the fungal diversity of unresolved taxa from the provided environmental samples.
7. We also addressed some of the current hypotheses concerning the origin and development of marine fungi with their rationales. We suggest, the extant marine fungi were originated from freshwater and subsequently moved from land and, because of the strong natural selection, the species evolved and adapted to the sea.

## Author Contributions

VK designed this study with VS and G-FH. VK collected the literature and prepared the first draft of the manuscript. KT and VS critically revised the manuscript. J-JH and X-YL provided their valuable insights to the manuscript. All authors read and approved the final version of the manuscript.

## Conflict of Interest

The authors declare that the research was conducted in the absence of any commercial or financial relationships that could be construed as a potential conflict of interest.

## Publisher’s Note

All claims expressed in this article are solely those of the authors and do not necessarily represent those of their affiliated organizations, or those of the publisher, the editors and the reviewers. Any product that may be evaluated in this article, or claim that may be made by its manufacturer, is not guaranteed or endorsed by the publisher.
